# *Cnestusquadrispinosus*, a new species of xyleborine ambrosia beetle from Thailand and Borneo (Coleoptera, Curculionidae, Scolytinae, Xyleborini)

**DOI:** 10.3897/zookeys.795.28384

**Published:** 2018-11-05

**Authors:** Wisut Sittichaya, Roger A. Beaver

**Affiliations:** 1 Department of Pest Management, Faculty of Natural Resources, Prince of Songkla University, Had Yai, Songkhla, 90112, Thailand Prince of Songkla University Songkhla Thailand; 2 161/2 Mu 5, Soi Wat Pranon, T. Donkaew, A. Maerim, Chiangmai 50180, Thailand Unaffiliated Chiangmai Thailand

**Keywords:** Ambrosia beetles, Brunei, Cnestus, new species, Sabah, Thailand

## Abstract

A new species, *Cnestusquadrispinosus*, is described from Thailand, Brunei Darussalam, and East Malaysia (Sabah). It is compared to three related species of *Cnestus* which lack a mycangial tuft of hairs on the pronotum, and have an impressed elytral declivity.

## Introduction

The genus *Cnestus* Sampson was erected for a single species, *Cnestusmagnus* Sampson, 1911, from Sri Lanka (Sampson 1911). Nunberg (1972) in a review of and key to the genus included 12 species. Wood (1986) included *Cnestus* in the tribe Xyleborini, and keyed it from other genera in the tribe. He considered that there are about 17 species in the Oriental region and Japan (Wood 1986). The catalog of Wood and Bright (1992) includes 21 species. However, some of these are synonyms or need to be transferred to other genera (Smith SM, Beaver RA, Cognato AI, unpublished data). Dole et al. (2010) discussed the phylogenetic relationship of the genus to other xyleborine genera, and Dole and Cognato (2010) transferred eleven more species to the genus, thereby extending its range to the Neotropical region. Hulcr and Cognato (2013) diagnose and describe the characters of the genus, and key 4 species found in New Guinea. Currently, we recognise 25 species in the Old World and Pacific islands, and 4 species in the Neotropical region. One Oriental species, *Cnestusmutilatus* (Blandford), has been imported to North America (Schiefer and Bright 2004, Gomez et al. 2018). All species are inbreeding and fungus-farming ambrosia beetles (Wood 1986, Hulcr and Cognato 2013). Recent collecting by the senior author in the far South of Thailand has revealed a new species, which also occurs on the island of Borneo.

## Materials and methods

Specimens were collected using ethanol baited traps in the lowland tropical rain forest of the Hala-Bala Wildlife Sanctuary, Narathiwat province, Thailand. Specimens from Brunei Darussalam and East Malaysia (Sabah) were among material sent to RAB for identification by the Natural History Museum, London, and Dr. A. Floren. Photographs were taken with a Canon 6D digital Camera with a Canon MP-E 65mm Macro Photo Lens (Canon, Tokyo, Japan) and StackShot-Macrorail (Cognisys Inc, MI,USA) The photos were then combined with Helicon Focus 6.8.0. (Helicon Soft, Ukraine), all photos were improved with Adobe Photoshop CS6 (Adobe Systems, California, USA).

### Abbreviations used for collections

**NHMUK** Natural History Museum, London

**NHMW** Naturhistorisches Museum Wien, Wien

**PSUZC** Prince of Songkla University Zoological Collection, Songkhla

**THNHM** Natural History Museum of the National Science Museum, Thailand

**RAB** Private collection of Roger A. Beaver, Chiang Mai

**WST** Private collection of Wisut Sittichaya, Songkhla

## Taxonomy

### Cnestus quadrispinosus sp. n.

Taxon classificationAnimaliaColeopteraCurculionidae

http://www.zoobank.org/FB70D798-BA22-425F-8281-AEC901EA88F9

Figure 1A-F

#### Type material.

Holotype: female, THAILAND, Hala-Bala Wildlife Sanctuary, Narathiwat Province, lowland tropical rainforest, 5°47’44’’N, 101°50’07’’E, 01.iii.2014, ethanol baited trap (W. Sittichaya) (NHMW). Paratypes: 12 females, same data as holotype (NHMW, 2; PSUZC, 2; THNHM, 2; RAB, 1; WST, 5). BRUNEI, Kuala Belalong FSC, E115°7', N4°34', Dipterocarp forest, *Dryobalanopsbeccarii*, Aerial F[light]I[ntercept]T[rap] 3, 220m alt., 30.v.[19]91, N. Mawdsley NM 178 (NHMUK, 1). [E. MALAYSIA], Sabah, Poring Spring, Lower montane mixed dipterocarp forest, *Xanthophyllumaffine*, Fog XA11/F1, 12.v.1992, A. Floren (RAB, 1).

#### Diagnosis.

The species is placed in *Cnestus* because it possesses the following combination of characters: body rather short and stout, with very sparse vestiture; antenna with four-segmented funicle (including pedicel), club truncate and flattened, its first segment covering the whole posterior surface; anterior margin of pronotum with two large, upcurved denticles, lateral margins of pronotum carinate, disc not asperate; scutellum flush with elytral surface; procoxae narrowly separated, intercoxal process spinelike, posterocoxal process not swollen; protibiae obliquely triangular, widest about one-fourth from apex, outer margin with 6‒7 denticles in apical half, posterior face not tuberculate.

The species is distinguished from all other species of *Cnestus* by the large spine on interstriae III at the upper margin of the elytral declivity, and a second large spine on interstriae V on the lateral margin of the declivity. The species belongs to a small species group which lack a mycangial tuft of hairs at the base of the pronotal disc, and in which the elytral declivity is broadly impressed. The majority of *Cnestus* species have a mycangial tuft indicating the presence of a mesonotal mycangium, and a convex elytral declivity.

#### Description.

*Female*. Length 4.25 mm (paratypes 3.45-4.50 mm), 2.30 times longer than wide (paratypes 2.20-2.56 times), body stout, shining, bicoloured, head dark brown to black, pronotum dorsally entirely black, laterally brown to dark brown, at least anterior part of elytral disc yellowish brown to dusky brown, area of paler colour varying individually from a small area at base of elytra to whole disc, remainder of elytral disc and declivity dark brown to black; ventrally yellowish brown, femora pale, tibiae dark brown, antennae and tarsi brown.

*Head*. Frons moderately convex, shining, with an indistinct small, smooth median swelling above epistoma, and a broader slightly raised smooth area towards vertex, lower part with scattered elongate rugae, arranged subconcentrically around lower swelling, upper part with fine punctures laterally; vestiture of fine hairs of variable length, longer and denser on lower part of frons; epistoma with dense brush of stiff, yellowish setae. Eyes shallowly emarginate at antennal insertion, lower portion distinctly larger. Antenna type 1 (Hulcr et al. 2007), scape long and slender, weakly spatulate, pedicel cup-shaped, funicle 3-segmented, the segments successively wider, antennal club large, subcircular and very flat, segment I covering posterior side, segment II corneous, visible only on anterior side.

*Pronotum*. Near type 7 in dorsal and lateral view (Hulcr et al. 2007), approximately as long as wide (holotype 1:1.03; paratypes 1:0.86-1.09), basal margin raised, shallowly, broadly emarginate; sides weakly curved in basal half, widest at about middle, more strongly curved anteriorly, anterior margin projecting over head with two large upcurved asperities at apex; anterior slope convex, armed with robust, pointed asperities anteriorly, the asperities becoming more transverse, more closely spaced and lower towards summit in middle; disc weakly shining, weakly reticulate, finely punctured, the punctures more closely spaced in the Thai than in the Bornean specimens, finer and sparser posterolaterally; posterolateral margin acutely carinate from basal margin to middle of pronotum; vestiture on pronotal slope sparse, with long erect setae, disc glabrous, without mycangial tuft.

*Scutellum*. Small, flat, semicircular, impunctate.

*Elytra*. Holotype 1.13 times longer than wide (paratypes 1.05-1.33), 1.29 times longer than pronotum (paratypes 1.14-1.36), bases transverse, carinate from scutellum to humerus, a small longitudinal swelling at humerus; sides subparallel in basal two-thirds, then gradually rounded to apex; elytral disc shining, convex, striate-punctate, strial punctures fine, moderately dense, interstrial punctures uniseriate, coarser and a little more closely placed than those on striae, both sets of punctures more closely placed in Thai than in Bornean specimens, disc with a few long and fine yellowish interstrial setae; declivity commencing at about middle, steeply sloping, declivital face quite strongly, broadly, impressed, sub-shiny, the margins carinate from the apex to interstriae VII; upper margin of the declivity with a small spine on interstriae II and a much larger, posteriorly directed spine on interstriae III, another large spine of similar size on interstriae V at about mid-height of declivity, small spines or granules may also be present between these large spines on declivital margins; striae I and II impressed on the upper part of declivity, interstriae II and III much widened on declivity, almost flat, each with a row of widely separated granules or small spines bearing very long, fine hairs posteriorly.

*Legs*. Procoxae narrowly separated, anterocoxal process narrow, spine-like, posterocoxal process not swollen; protibia obliquely triangular, widest about one-fourth from apex, outer margin with 6‒7 denticles in apical half, posterior face weakly convex, not tuberculate. Meso- and meta-tibiae more evenly rounded with 10-11 denticles on outer margin.

*Male.* Unknown.

**Figure 1:**
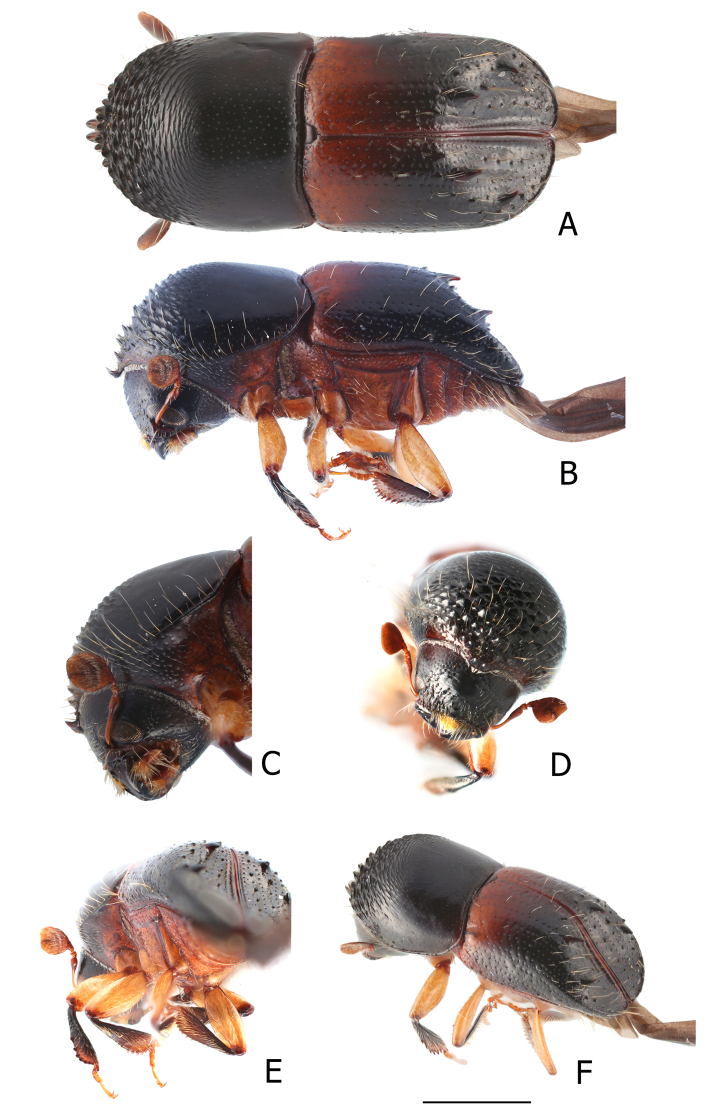
*Cnestusquadrispinosus* sp. n. A Dorsal view, B Ventro-lateral view, C Antenna, D front, E pro-, meso-, and meta-tibia, F posterolateral view. Scale bar: 2 mm.

#### Etymology.

The Latin name is an adjective derived from the four (*quatuor*) spines (*spinae*) on the elytral declivity.

#### Distribution.

Brunei Darussalam, East Malaysia (Sabah), Thailand.

#### Host plants.

Unknown.

## Discussion

*Cnestusquadrispinosus* is clearly related to three other species: *C.bicornis* (Eggers, 1923), *C.bicornioides* (Schedl, 1952), and *C.triangularis* (Schedl, 1975). All four species lack a mycangial tuft of hairs at the base of the pronotum, and have broadly impressed elytra. This combination of characters distinguishes them from all other species of *Cnestus*: *C.quadrispinosus* is easily distinguished from the other three species by the presence of two pairs of large spines on the declivity. *Cnestusbicornis* is distinguished by the more elongate, parallel-sided pronotum, and the fine, sparse punctures of the pronotal disc. We have been unable to find characters that will reliably separate *C.bicornioides* and *C.triangularis*, and suspect that the two species should be synonymised. However, further studies of the species are needed.

### Key to the species of *Cnestus* lacking a pronotal mycangial tuft and with impressed elytral declivity

**Table:** 

1	Interstriae III and V each with a single strong tooth on upper and lateral margins of elytral declivity respectively. Thailand, Brunei, East Malaysia	***C.quadrispinosus* sp. n.**
‒	Interstriae III and V without large teeth on declivity	2
2	Pronotum parallel-sided, 1.25 times longer than wide. Pronotal disc very finely, very sparsely punctured. Length 2.2–2.6 mm. Indonesia (Java, Sumatra), Philippines	***C.bicornis* (Eggers)**
–	Pronotum with weakly curved sides, 1.0–1.1 times longer than wide. Pronotal disc more densely and coarsely punctured. Length 2.7‒3.7 mm. China (Yunnan), India (Andaman Is., Bengal), Papua New Guinea, Philippines, Thailand, West Malaysia	***C.bicornioides* (Schedl),= ? *C.triangularis* (Schedl)**

In the majority of *Cnestus* species, the females possess a mesonotal mycangium used to transport the ambrosial fungus on which the larvae feed (Stone et al. 2007, Hulcr and Cognato 2013). Its presence is indicated by a tuft of hairs at the base of the pronotum. It is not known whether the four species have lost this mycangium, or whether the mycangium is situated elsewhere in the body, most probably in the head. If the mycangium has been lost, the species may well be mycocleptic (Hulcr and Cognato 2010). In mycocleptic species, the female starts its gallery close to galleries of other ambrosia beetles. The fungus established by the ‘host’ species grows in the galleries of the mycoclept which consequently does not need to transport its own ambrosia fungus, and lacks mycangia (Hulcr and Cognato 2010).

## Supplementary Material

XML Treatment for Cnestus quadrispinosus

## References

[B1] Dole SA Cognato AI (2010) Revision of *Xylosandrus* Reitter (Curculionidae: Scolytinae). Proceedings of the California Academy of Science 61: 451–545.

[B2] Dole SA Jordal BH Cognato AI (2010) Polyphyly of *Xylosandrus* Reitter inferred from nuclear and mitochondrial genes (Coleoptera: Curculionidae: Scolytinae). Molecular Phylogenetics and Evolution 54: 773‒782. 10.1016/j.ympev.2009.11.011 19925873

[B3] Eggers H (1923) Neue indomalayische Borkenkäfer (Ipidae). Zoologische Mededelingen 7: 129‒220.

[B4] Gomez D Rabaglia RJ Fairbanks KEO Hulcr J (2018) North American Xyleborini north of Mexico: a review and key to genera and species (Coleoptera, Curculionidae, Scolytinae). ZooKeys 768: 19‒68. https://doi. org/10.3897/zookeys.768.24697 PMC6019436 29955211

[B5] Hulcr J Cognato AI (2010) Repeated evolution of theft in fungus farming ambrosia beetles. Evolution 64: 3205–3212. 10.1111/j.1558-5646.2010.01055.x 20633043

[B6] Hulcr J Cognato AI (2013) Xyleborini of New Guinea: A Taxonomic Monograph. Thomas Say Publications in Entomology, Entomological Society of America, Annapolis, 176 pp.

[B7] Hulcr J Dole SA Beaver RA Cognato AI (2007) Cladistic review of generic taxonomic characters in Xyleborina (Coleoptera: Curculionidae: Scolytinae). Systematic Entomology 32: 568‒584. 10.1111/j.1365-3113.2007.00386.x

[B8] Nunberg M (1972) Die Gattung *Cnestus* Sampson (Coleoptera, Scolytidae). Annales Zoologici, Warszawa 29: 473–478.

[B9] Sampson FW (1911) On two new wood-boring beetles. Annals and Magazine of Natural History, Series 8, 8: 381–384. 10.1080/00222931108693046

[B10] Schedl KE (1952) Fauna Philippinensis VIII. Philippine Journal of Science 80: 363‒371.

[B11] Schedl KE (1975) New Scolytidae and Platypodidae from Papua and New Guinea IV. Annalen des Naturhistorischen Museums in Wien 79: 337‒399.

[B12] Schiefer TL Bright DE (2004) *Xylosandrusmutilatus* (Blandford), an exotic ambrosia beetle (Coleoptera: Curculionidae: Scolytinae: Xyleborini) new to North America. The Coleopterists Bulletin 58: 431‒438. 10.1649/760

[B13] Stone WD Nebeker TE Monroe WA MacGown JA (2007) Ultrastructure of the mesonotal mycangium of *Xylosandrusmutilatus* (Coleoptera: Curculionidae). Canadian Journal of Zoology 85: 232‒238. 10.1139/z06-205

[B14] Wood SL (1986) A reclassification of the genera of Scolytidae (Coleoptera). Great Basin Naturalist Memoirs 10: 1–126.

[B15] Wood SL Bright DE (1992) A catalog of Scolytidae and Platypodidae (Coleoptera), Part 2: Taxonomic index. Great Basin Naturalist Memoirs 13: 1–1553.

